# Epidemiology and injury trends in the National Basketball Association: Pre- and per-COVID-19 (2017–2021)

**DOI:** 10.1371/journal.pone.0263354

**Published:** 2022-02-10

**Authors:** Lorena Torres-Ronda, Ignacio Gámez, Sam Robertson, José Fernández

**Affiliations:** 1 Spanish Basketball Federation, Madrid, Spain; 2 Institute for Health and Sport, Victoria University, Melbourne, Australia; 3 BBVA Advanced Analytics Department, Madrid, Spain; 4 School of Behavioral and Health Science, Australian Catholic University, Melbourne, Australia; Universidad Autonoma de Madrid, SPAIN

## Abstract

**Purpose:**

The aim this study was to provide an epidemiological injury analysis of the National Basketball Association, detailing aspects such as frequency rate, characteristics and impact on performance (missed games), including COVID-19 related and non-related injuries.

**Methods:**

A retrospective study was conducted from the 2017–18 to 2020–2021 season. Publicly available records from the official website of the National Basketball Association were collected, including player’s profiling data, minutes played per game until the injury occurred, unique injuries and injury description [location (body area), diagnosis (or mechanism)], and missed games due to injury.

**Results:**

A total of 625 players and 3543 unique injuries were registered during the period analyzed. There was an increased incidence of missed games and unique injuries ratios, from 2017–18 until 2020–21, even when excluding COVID-19 related cases. The main body areas of injuries corresponded to lower body injuries, specifically knee, ankle and foot. The tendon/ligament group, for both games missed and unique injuries, showed the higher ratios (1.16 and 0.21, respectively), followed by muscle (0.69 and 0.16, respectively) and bones (0.30 and 0.03, respectively). Irrespective of season, the higher percentage of unique injuries occurred in the group of players playing in the 26–35 minutes, followed by the 16–25 minutes played. Guards showed the highest injury ratios compared to other playing positions. Most injuries and missed games due to injury occurred from mid-season to the end of the regular season. The majority of both injuries and missed games were concentrated in the two central experience groups (from 6 to 15 years).

**Conclusions:**

Despite previous efforts to better understand injury risk factors, there has been an increase in unique injuries and missed games. The distribution by body area, type of injury, when they occurred, minutes played and outcomes by play position, age a or years of experience vary between season and franchises.

## Introduction

The National Basketball Association (NBA) league is one of the five major professional sports leagues in North America (United States of America, Canada), along with Major League Baseball (MLB), National Football League (NFL), National Hockey League (NHL) and Major League Soccer (MLS). These leagues are the highest-level team sport competitions in those countries. The NBA is considered the premier basketball league in the world, with considerable domestic and international audiences. Furthermore, NBA players lead the average annual salaries when compared with the other professional sport major leagues worldwide (statista.com). Consequently, player health, their availability to train and compete, along with subsequent performance are some of the most important aspects for organizations to focus on.

Basketball is a stochastic contact sport where both, teammates and opponents must share a relatively small court, compared to other open field sports, and involves significant levels of technical, tactical, physical and mental requirements [1, 2]. NBA teams must navigate through a dynamic and complex structure of competition with a high density of fixtures throughout the season [3]. This can include participation in between two and five games in seven days, compressing consecutive games in two days, or ‘back-to-back’, (B2B), and having to travel across up to three time zone changes regularly. Players and teams also experience internal (e.g., pressure to win) and external (e.g., media commitments) pressures, which have the potential to compromise health and increase injury risk. A range of initiatives have been implemented by teams and the league in order to minimize this risk. This includes the league reducing the number of B2Bs [4], teams improving travel efficiency and adding more medical personnel to the staff. However there is still today a subjective perception of a lack of understanding of why certain injuries still occur so frequently (fortuitous and more preventable, such as soft tissue or stress injuries). This has led to an increased priority on examining how to minimize the risk of those injuries that exhibit aspects that may be somewhat controllable on the part of the player, league or staff.

There have been studies documenting the epidemiology and incidence of injuries in basketball players. The lower body (e.g., ankle, knee, hip), shows the most frequently injured areas [5]. Ankle sprains have been reported to be the most common injury across all levels of basketball participation for both genders [6–9], representing the 25.8% in NBA players in 2019 [10]. Previous studies estimated an incidence rate of 1.0 to 5.2 ankle injuries per 1000 person-hours in basketball with sprains accounting in 2007 for approximately 90% of these basketball-related ankle injuries [6]. Adductor, hip and groin injuries have been shown to account for 21.8% of all hip injuries in this population [11]. Patel et al. (2019) found 79 adductor injuries across 65 NBA athletes from 2009–2010 to 2018–2019 season, where 72 injuries were reported as strains (91%) and 7 reported as tears (9%) [12]. The impact on performance after Achilles tendon tears [13] anterior cruciate ligament injury, lumbar disc herniation [14] and metacarpal fractures [15] has also been reviewed. Regarding stress fractures/injuries, Khan et al. (2018) founded a total of 76 lower extremity bone stress injuries (n = 75 NBA players; from 2005 till 2015), where 55% involved the foot, 21.1% the ankle or fibula, 17.1% the tibia, and 6.6% either the knee or patella [16]. The most reported injury was a stress fracture to the fifth metatarsal (18.4%) followed by other stress fractures to the foot (14.5%). While body area location has been amply studied in basketballers, there is a lack of analysis in regard to the type (diagnosis, mechanism) of injury in professional NBA players. Teramoto et al. (2017) reported sprain/hyperextension as the most common type of injury (33.5%), followed by superficial injuries (16.6%), strain (12.8%) and irritation/soreness/swelling (12.7%).

There is a growing concern about injury incidence and performance decrement in the NBA as a result of the high density of games in the season [10]. With the advent of COVID-19 the league had to add modifications to the 2020–21 season schedule, resulting in an increase of game density again, however, there is a lack of analysis and understanding of the injury incidence since the COVID-19 pandemic impacted the competition in 2020.

The aim of the present study was to provide an epidemiological retrospective analysis for the last four NBA seasons analyzed (2017–18 to 2020–21), detailing aspects such as frequency rate, characteristics (body area, diagnosis) and impact on performance (missed games) in the NBA. Furthermore, new insights into the effect COVID-19 on injuries are also be presented.

## Methods

### Study design

A retrospective study was conducted from the 2017–18 to 2020–2021 season, including all 30 NBA teams. Institutional Review Board approval was not a requirement for this investigation, as we only included publicly available data for analysis, collected via web scraping techniques. Additionally, the data only contains non-identifiable information about participants.

### Data source

Publicly available records from the official website of the National Basketball Association (NBA.com) were collected. After each game, the NBA reports the Official Scorer’s Report (Final Box), also known as Box Score (portable document format; PDF). This document is the official final game box score which includes the official inactive players that teams must provide for legal purposes before the game starts. It includes inactive players (for home and visiting team), team’s name, player’s last name, and injury area and cause (mechanism, diagnosis, reason) [e.g.1: player Last Name (Injury/Illness—Left Ankle; Sprain); e.g.2: player Last Name (Health and Safety Protocols); e.g.3: player Last Name (Injury/Illness—Left Knee; Meniscus Surgery Recovery)]. It also includes ‘Did Not Play’ (DNP; not always specifying if by ‘coach decision’ or ‘injury related’). In order to use only the official NBA league’s public information, no other websites, online sites or press release platforms were included. Despite having electronical access to earlier seasons, we decided to only include data from the season 2017–18 onward, due to many games missing data and missed injury data in the Box Score prior to 2017.

### Data definitions

**Fig 1:**
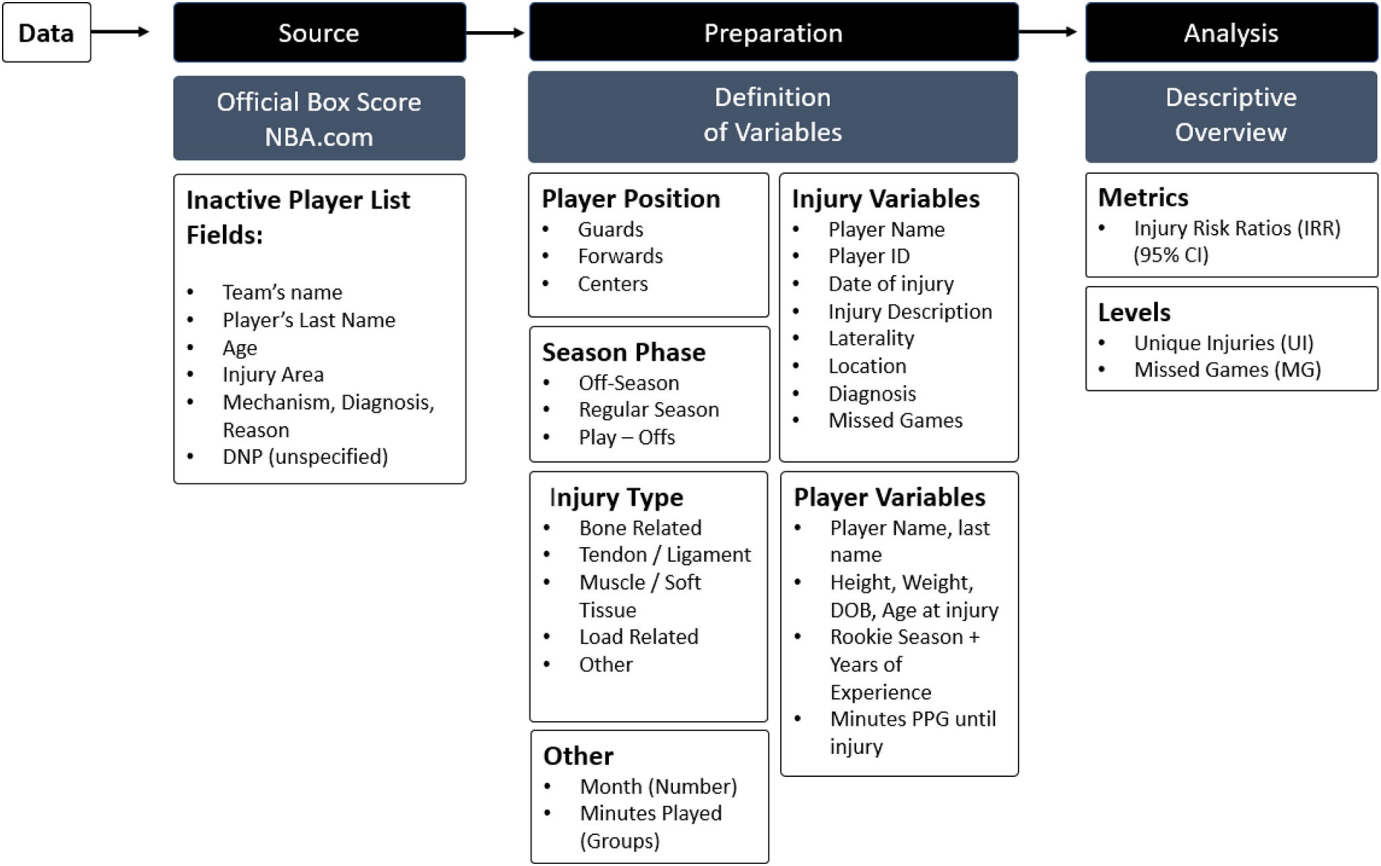
Data schema.

The player database (Fig 1) included player name and last name, playing position, height, weight, date of birth, player’s age at the time of the injury, rookie season, years of experience (calculated as the difference between the year of the rookie season and the latest season analyzed), and minutes played per game (MP) until the injury occurred. For an injury to be recorded in the database, the injury must be published in the official Boxes Score of the game (PDF). For each injury (aka unique injury; UI), the following data was registered: player name and player identification (ID), game date and game ID, date of injury, injury description, laterality [when available (i.e., right, left)], location (body area from now on), and diagnosis (or mechanism). Injury categories were mutually exclusive. An injury ID was assigned to every unique injury (UI), same location, same diagnosis, for a player, until the player played again in an official game. For every player-injury ID, the variable missed games (MG), as a resulted in at least one missed NBA game (inactive, DNP) after the first registration of an injury (UI), was calculated. If the remainder of the season was lost, the days were counted from the date of the first game lost and the date of the last game played that season.

The NBA provides to each team an injury report which team only data at the end of the season (the annual team-specific injury report). The authors of this study used the location (body area) and diagnosis of injuries as a reference, and map the body areas, diagnosis (mechanism or causes of the injury) with the current study database. Moreover, based on the same report, and previous literature, unique injuries were also analyzed and categorized as follows: i) bone related (bone-stress, bone-fracture, bone-dislocation); ii) tendon/ligament related (general soreness, sprain, tendinopathy, anterior cruciate ligament (ACL), other meniscus, tendon/ligament-bursitis, tear, tendinitis, ligament damage, medial collateral ligament (MCL), tendinosis, impingement, subluxation, tendinosis); iii) muscle related (strain, general soreness, tear, tightness, inflammation, pain, muscle-stress); iv) load related (management, load-stress related); and v) others (bursitis, dislocation, disruption, edema, effusion, fasciitis, impingement, inflammation, laceration, medical, muscle strain, nerve irritation/damage, neuropathy, NSBP, procedure, subluxation).

The season phase (following common teams classification, and operational reasons) at the time of injury was classified as follows: i) Preseason (1^st^ official pre-season game), Regular season (RS; from the 1^st^ official game until the last official game), and Playoffs (PO; from the 1^st^ playoff games until the last game played). The COVID-19 lockdown occurred form March 11^th^ 2020 until the Bubble, July 22^nd^ 2020 (first exhibition scrimmages). Offseason injuries and those that are not reported in the official Box Score (PDF) were not included. Players were divided in three categories as per their playing position: i) guards (guard, guard-forward), ii) forwards (forward, forward-guard, forward-center), and centers (center, center-forward).

### Data analysis

The collection of the data Box Score (PDF) was performed using a Python using Python 3.6.3::Anaconda, Inc. (Python, Netherlands). A descriptive analysis was performed for all variables of interest, including frequency (counts and percentage) and means. In order to standardize the number of injuries by season, with different number of total games each season, Injury Rate Ratios (IRRs), with associated 95% CIs, were calculated. The IRRS were calculated as follows; for each unique injury (corresponding to a unique player), the number of missed games was divided by the number of games of his team (including pre-season games and playoff games). The number of regular season games don’t vary between teams; however, the number of pre-season and playoff games was specific for each team (and player). If a player was transferred and missed a few games due to injury with the former team (team A) and a few games with the arriving team (team B), he had two IRRs, one corresponding to the number of games of the first team (A) and another for the number of games of the second team (B). Comparisons between season were analyzed using two one-sided test (TOST) procedure to test the equivalence and reject the presence of a smallest effect size of interest (SESOI). Standardized effect sizes were set at Cohen’s *d* raw mean difference. The descriptive and statistical analysis were performed using Microsoft Excel (Microsoft Corporation, WA, USA), and R programming language (v3.0.1) RStudio IDE (v1.4).

## Results

### Epidemiology

During the period of the analysis (2017–18 to 2020–21 seasons) a total of 625 players (unique ID) and 3543 unique injuries were registered. The number of games played, unique injuries, and missed games by injury (including COVID-19 related events), from 2017–18 until 2020–21 seasons are displayed in Table 1. The total number of games was lower in the 2020–21 season while the overall density was higher than in previous seasons due to a more congested schedule. As a result, a higher number of absolute unique injuries and games missed due to injury can be seen. The 2020–21 season showed a higher UI% compared to previous seasons (Table 1). When comparing year to year, results showed and upward tendency for unique injuries, however, the variation was small to be significant. The number of missed games showed a significant increment from season 2019–20 to 2020–21 (Table 2).

**Table 1:** Descriptive data for the seasons analyzed, missed games and unique injuries by type of season phase.

	**2017–18**			**2018–19**			**2019–20**						**2020–21**		
**Games per team, RS (n)**	82			82			72*						72		
**Regular season days (n)**	176			176			166**						145		
**Density of games, RS (games/RS days)**	0.47			0.47			0.46						0.50		
**Total Games, RS+PO (n)**	1390			1391			1254						1214		
**Unique Injuries (UI)**	821			901			868						953		
**UI % difference compared to 2020–21**	-33%			-21%			-13%						-		
**Number of injured players**	306			339			356						392		
**Missed games due to injury (MG)**	4898			4974			4356						5228^#^		
	**PS**	**RS**	**PO**	**PS**	**RS**	**PO**	**PS**	**RS**	**Bubble**				**PS**	**RS**	**PO**
									**Total**	**PS**	**RS**	**PO**			
UI Total (n)	10	788	23	79	788	34	22	661	185	8	131	46	7	907	39
UI COVID-19 related (n)									1				2	154	2
MG Total (n)	14	4759	125	112	4669	193	25	3781	550	10	317	223	13	4999	216
MG COVID-19 related (n)									1				5	637	4

RS: Regular season phase. PO: Playoffs phase. UI: Unique injuries. MG: Missed games.

*:72 including the 8 games in the Bubble.

**: includes regular season days until the pause of the season (in March) and the Bubble regular season days (starting in July).

^#^: significant difference with previous season.

**Table 2:** Comparisons of missed games and unique injuries ratios between seasons.

	Unique Injuries				Missed Games			
	Mean (SD)	CV	T-Test (U-L)	p	Mean (SD)	CV	T-Test	p
2017–18	0.29 (0.11)	0.36	-1.33 (-1.6; -1.1)	0.19	1.77 (0.67)	0.38	-0.18 (-0.51; 0.14)	0.85
2018–19	0.32 (0.10)	0.30	-0.97 (-1.25; -0.70)	0.34	1.78 (0.64)	0.36	0.23 (-0.10; 0.55)	0.82
2019–20	0.34 (0.12)	0.36	-1.98 (-2.25; -1.70)	0.05	1.76 (0.70)	0.38	-3.08 (-3.46; -2.71)	0.004^#^
2020–21	0.40 (0.13)	0.34			2.18 (0.75)	0.34		

SD: standard deviation. CV: coefficient of variation. U: upper bound. L: lower bound. The T-Test compares the ratios for the season against following season.

^#^: significant difference (p<0.05).

Season 2018–19 showed a greater number of pre-season injuries compared to the other seasons; on the other side, 2020–21 showed a higher number of unique injuries and games missed during the regular season.

**Fig 2:**
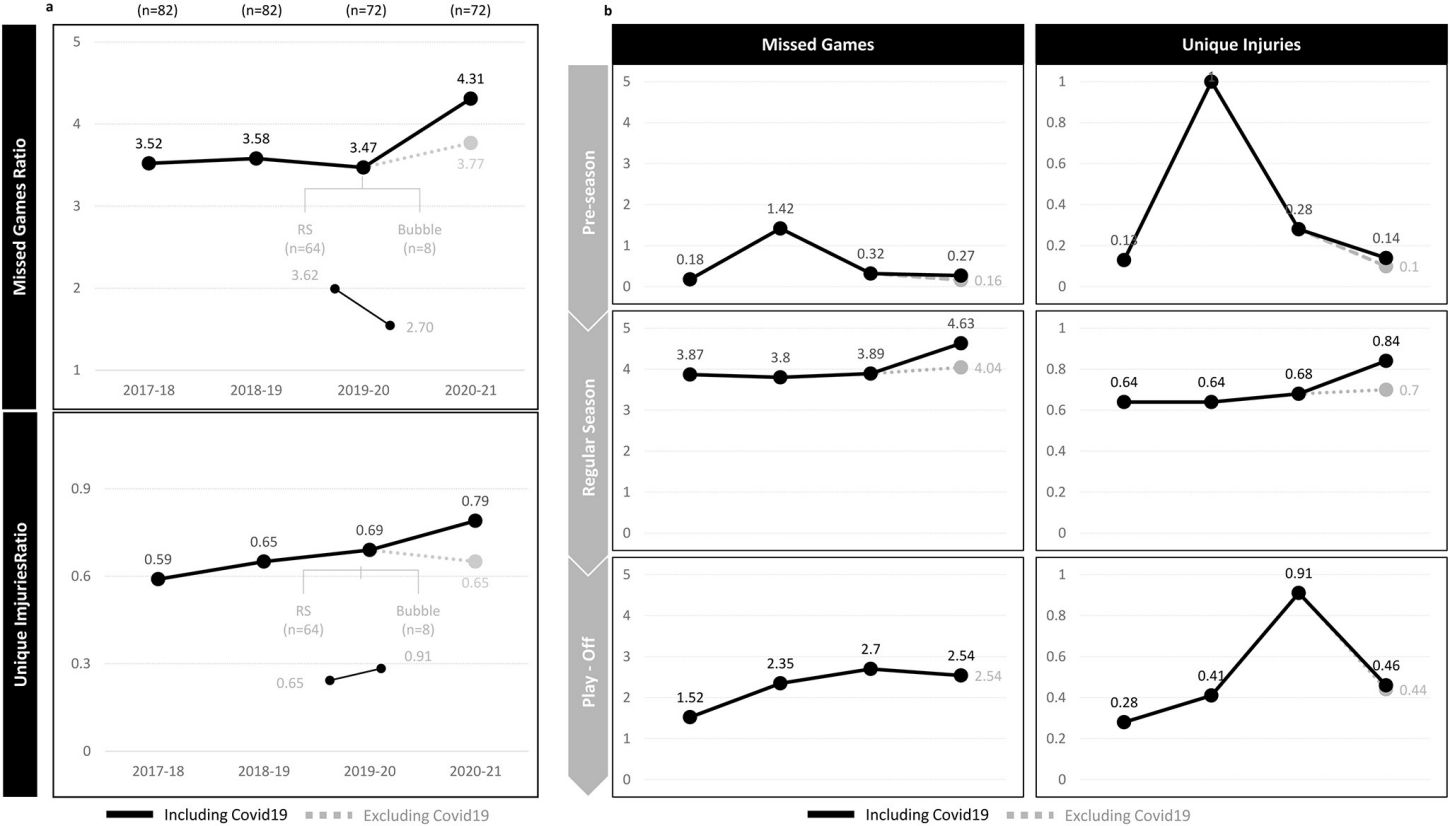
Missed games and unique injuries ratios by season, by season phase, including and excluding COVID-19 related injuries. n: number of game per team.

The results showed an increased tendency in both, missed games by injury and unique injuries (number of injuries) ratios, from 2017–18 until 2020–21, even when excluding COVID-19 related cases (Fig 2a). When splitting 2019–20 in RS and Bubble, results showed a decrease of IRR for missed games and unique injuries during the Bubble phase, which reflected a slightly lower ratio of missed games by injury in 2019–20 (Fig 2a).

### Season-phase

When segmenting the analysis by phase of the season, results showed higher IRR for both, missed games and unique injuries during the 2018–19 pre-season phase. During the regular season, there was a higher number of games missed during 2020–21, even when excluding COVID-19 related (Fig 2b). When looking at the post-season, results showed a decrease in the ratio of missed games in 2020–21 compared to the previous seasons however, this ratio was still higher than in seasons prior 2019–20 (Fig 2b).

### Body area

**Fig 3:**
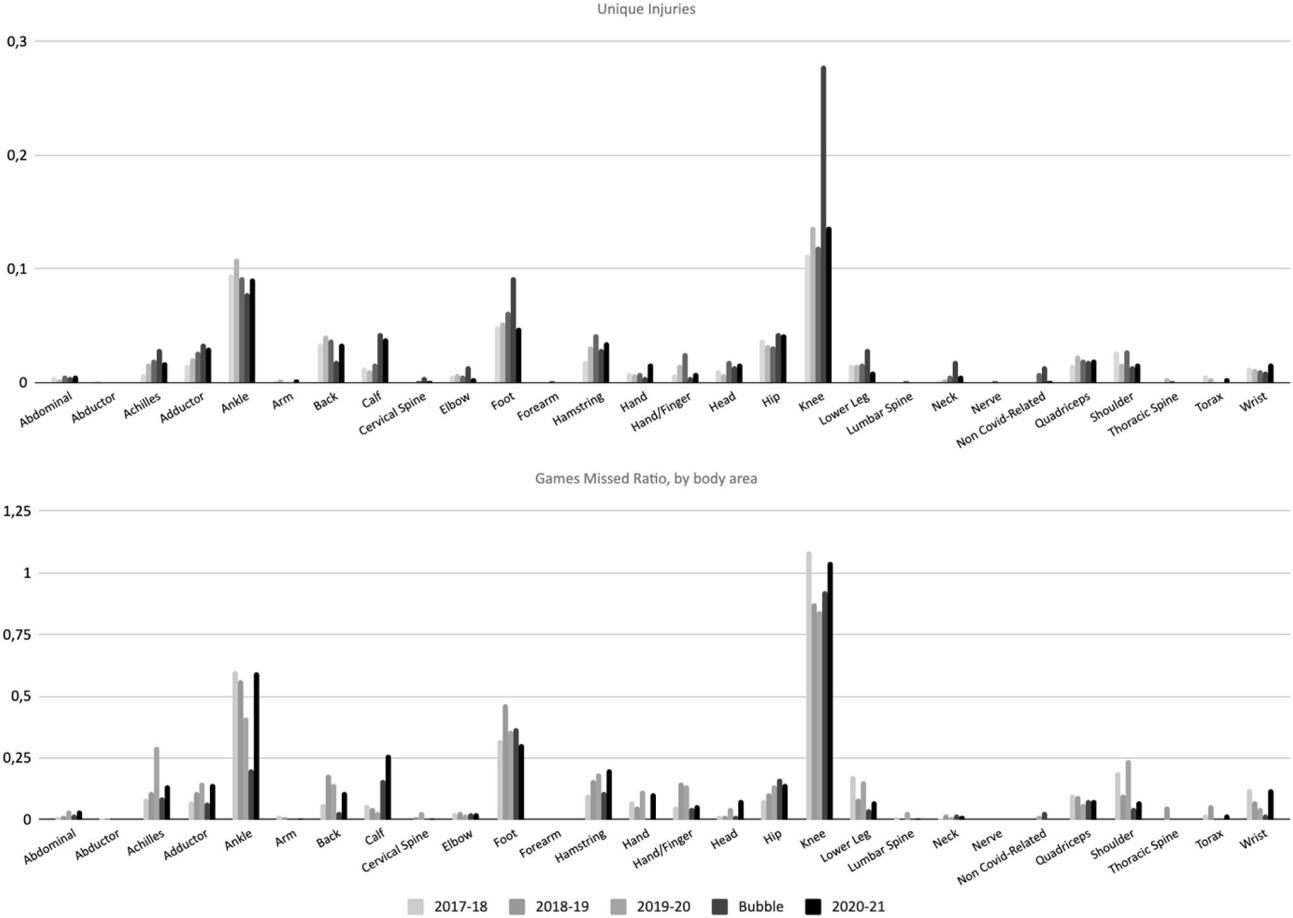
Missed games and unique injuries ratios by body area and for the four seasons analyzed.

**Fig 4:**
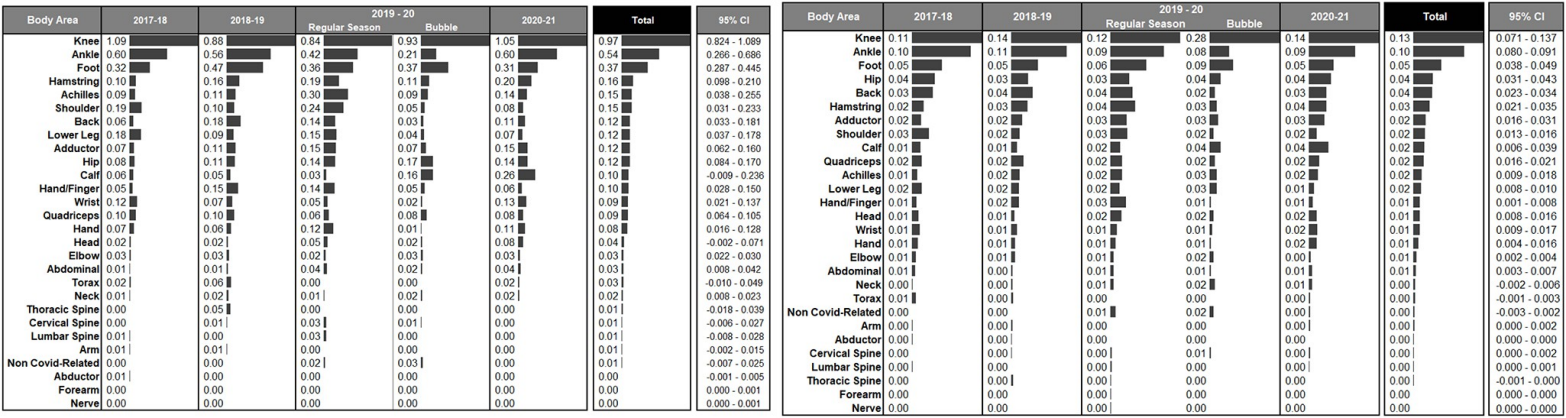
Missed games and unique injuries ratios by body area, total and 95% confidence interval.

When reviewing the area of injury, lower body injuries, specifically knee, ankle and foot seem to be more affected, for both missed games and unique injuries (Fig 4). The highest IRR of unique knee injuries occurred during the Bubble period (2.80), however, the highest IRR of games missed due to knee injuries occurred during the 2017–18 and 202–21 seasons. The highest IRR of unique ankle injuries occurred during the 2018–19 season; despite showing a decreased trend of IRR from 2017–18 to 2019–20, the number of games missed increased again in 2020–21. Similarly, the IRR of unique foot injuries showed the highest value during the Bubble however, the ratio of missed games was higher during the 2018–19 season. Fig 3 shows the unique injuries and missed games ratios by body area per season to display trends by season.

### Type of injury

When classifying the injuries grouped by type (i.e., bone, muscle, tendon/ligament, load-related, or COVID-19 related), the tendon/ligament group for both, missed games and unique injuries, showed the highest ratios (1.16 and 0.21, respectively), followed by muscle (0.69 and 0.16, respectively) and bone (0.30 and 0.03, respectively) (Table 3). The specific mechanism of injury (diagnosis) that caused a higher IRR of missed games was medical procedures (0.70), followed by sprain (0.52), general soreness (0.45), strain (0.35) and fracture (0.25). When paying attention to the season, 2020–21 showed the highest IRR for games missed by tendon/ligament injuries (1.47), while the highest IRR for unique tendon/ligament injuries occurred during the Bubble (0.47).

**Table 3:** Missed Games and unique injuries by type (clusters) of injury.

	Injury Type	2017–18	2018–19	2019–20	Bubble	2020–21	Total	95% CI
Missed Games Ratio	**Bone**	0.03	0.14	0.51	0.24	0.34	0.30	0.139–0.467
	**Muscle**	0.42	0.65	0.78	0.60	0.98	0.69	0.424–0.949
	**Tendon / Ligament**	1.08	1.09	1.01	1.03	1.47	1.16	0.901–1.369
	**Load**	0.15	0.06	0.09	0.08	0.05	0.09	0.039–0.134
	**Covid-Related**	0.00	0.00	0.00	0.01	0.53	0.12	-0.187–0.402
	**Other**	3.17	3.16	2.94	3.89	2.56	3.01	0.747–1.710
Unique Injury Ratio	**Bone**	0.03	0.01	0.06	0.03	0.03	0.03	0.010–0.055
	**Muscle**	0.11	0.17	0.18	0.22	0.19	0.16	0.126–0.220
	**Tendon / Ligament**	0.18	0.21	0.19	0.33	0.22	0.04	0.153–0.299
	**Load**	0.05	0.04	0.02	0.07	0.04	0.03	0.023–0.068
	**Covid-Related**	0.00	0.00	0.00	0.01	0.13	0.03	-0.045–0.099
	**Other**	0.22	0.22	0.18	0.26	0.19	0.21	0.178–0.249

CI: confidence interval.

### Type of injury by minutes played before injury occurred

**Fig 5:**
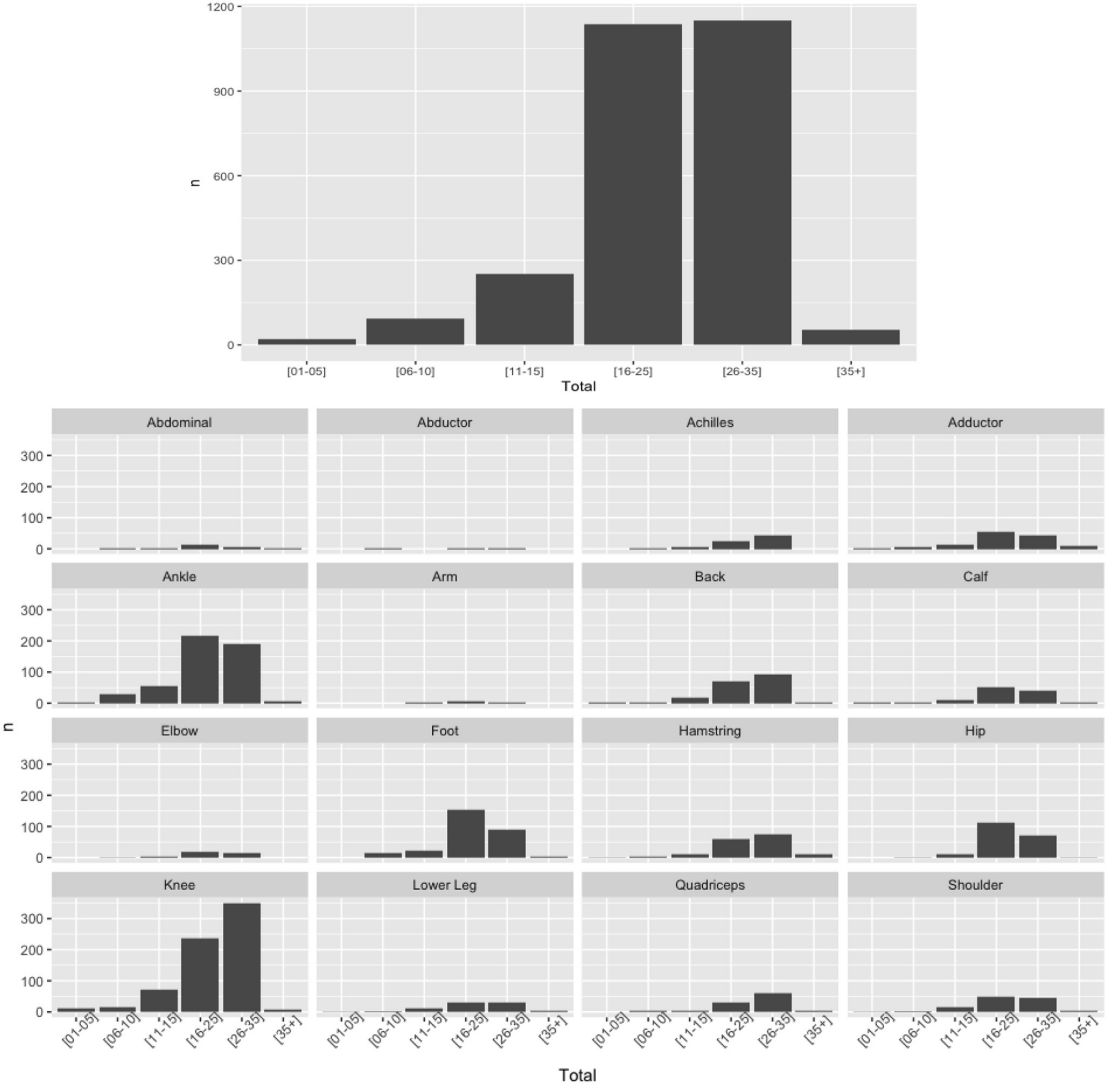
n: Number of game per team.

Irrespectively of the season, the higher percentage of unique injuries occurred in the group of players playing in the 26–35 minutes, followed by the 16–25 minutes played (MP) (Fig 5a; Table 4). For the 26–35 MP, for all the seasons analyzed, the higher percentage of UI corresponded to Load related injuries (52.9%, 53.6%, 63.4% and 68.2%, respectively for each season), followed by tendon/ligament injuries (42.9%, 46.8%, 51.5%, and 45.5%, respectively for each season). For the 16–25 MP group, the higher % of unique injuries varied depending on the season (Fig 5b; Table 4).

**Table 4:** Season % of unique injuries by type of injury and grouped by playing time.

Season	Type of Injury	[01–05]	[06–10]	[11–15]	[16–25]	[26–35]	[35+]	n	95% CI
2017–18	**Bone**	0.0%	5.6%	25.0%	36.1%	33.3%	0.0%	36	-0.92%–34.25%
	**Muscle**	2.6%	4.5%	12.1%	42.7%	33.8%	4.5%	157	-1.43%–34.76%
	**Tendon / Ligament**	0.8%	2.4%	12.6%	38.5%	42.9%	2.8%	247	-3.39%–36.72%
	**Load**	0.0%	0.0%	4.4%	42.7%	52.9%	0.0%	68	-8.93%–42.26%
	**Other**	3.9%	13.8%	17.5%	32.8%	30.6%	1.4%	1192	2.90%–30.43%
	**Total**	3.1%	10.5%	15.9%	35.0%	33.7%	1.8%	1700	1.33%–32.01%
2018–19	**Bone**	0.0%	5.0%	20.0%	40.0%	35.0%	0.0%	20	-2.01%–35.34%
	**Muscle**	0.4%	5.2%	11.4%	35.8%	44.5%	2.6%	229	-3.04%–36.37%
	**Tendon / Ligament**	2.5%	5.0%	11.5%	33.5%	46.8%	0.7%	278	-3.27%–36.60%
	**Load**	0.0%	1.8%	17.9%	21.4%	53.6%	5.4%	56	-4.39%–37.72%
	**Other**	2.9%	15.1%	15.9%	30.8%	32.9%	2.5%	1202	2.95%–30.39%
	**Total**	2.4%	11.7%	14.7%	31.7%	37.2%	2.3%	1785	1.21%–32.12%
2019–20	**Bone**	2.8%	7.0%	8.5%	62.0%	16.9%	2.8%	71	-7.25%–40.58%
	**Muscle**	0.0%	3.9%	8.7%	37.4%	48.3%	1.7%	230	-5.12%–38.45%
	**Tendon / Ligament**	0.4%	8.0%	10.2%	29.6%	51.5%	0.4%	274	-4.45%–37.78%
	**Load**	0.0%	0.0%	9.8%	19.5%	63.4%	7.3%	41	-8.54%–41.87%
	**Other**	2.3%	16.2%	16.7%	27.7%	35.9%	1.3%	1255	2.34%–31.00%
	**Total**	1.7%	12.8%	14.3%	30.3%	39.6%	1.4%	1871	0.50%–32.83%
2020–21	**Bone**	0.0%	12.9%	6.5%	35.5%	45.2%	0.0%	31	-3.47%–36.80%
	**Muscle**	1.8%	9.7%	6.2%	38.5%	36.7%	7.1%	226	-0.58%–33.91%
	**Tendon / Ligament**	4.3%	4.3%	7.8%	37.4%	45.5%	0.8%	257	-3.79%–37.12%
	**Load**	0.0%	0.0%	0.0%	20.5%	68.2%	11.4%	44	-11.22%–44.55%
	**Covid-Related**	4.5%	11.5%	12.1%	38.2%	30.6%	3.2%	157	1.56%–31.78%
	**Other**	5.2%	13.5%	7.6%	30.3%	40.8%	2.6%	1087	0.51%–32.83%
	**Total**	4.4%	11.2%	7.7%	32.9%	40.8%	3.1%	1802	-0.19%–33.52%

n: number of players.

### Playing position

The injury rate by playing position is displayed in Table 6. Guards showed the highest IRR compared to the other playing positions, for all the seasons analyzed, being 2020–21 and 2017–18 the seasons with the highest IRR for missed games (2.07 and 1.83, respectively), followed by forwards, whom showed a higher IRR of missed games in 2020–21 and 2019–20 (1.77 and 1.50 respectively). Both guards and forwards showed the highest number of unique injuries during the Bubble, followed by the 2020–21 season (Table 5).

**Table 5:** Missed games and unique injuries by playing position.

	Position	2017–18	2018–19	2019–20	Bubble	2020–21	Total	95% CI
Missed Games Ratio	**Guard**	1.83	1.58	1.58	1.39	2.07	1.75	1.362–2.017
	**Forward**	1.16	1.30	1.50	1.12	1.77	1.41	1.037–1.704
	**Center**	0.46	0.64	0.52	0.11	0.41	0.50	0.182–0.677
Unique Injury Ratio	**Guard**	0.24	0.27	0.28	0.43	0.38	0.29	0.217–0.419
	**Forward**	0.21	0.25	0.26	0.34	0.29	0.26	0.211–0.333
	**Center**	0.10	0.09	0.09	0.06	0.08	0.09	0.067–0.098

CI: confidence interval.

**Table 6:** Injury ratios by age group.

	AGE GROUP	2017–18	2018–19	2019–20		2020–21	Total	95% CI
				RS	Bubble			
Missed Games Ratio	**[19–24]**	0.0068	0.007	0.0062	0.005	0.0086	0.0018	0.0051–0.0084
	**[25–29]**	0.0082	0.0065	0.0072	0.0044	0.0082	0.0019	0.0049–0.0088
	**[30–34]**	0.005	0.0084	0.0099	0.0075	0.0082	0.0019	0.0056–0.0100
	**35+**	0.0035	0.0083	0.0043	0.0033	0.0093	0.0016	0.0022–0.0093
Unique Injuries Ratio	**[19–24]**	0.0009	0.001	0.001	0.0014	0.0013	0.0003	0.0009–0.0014
	**[25–29]**	0.0012	0.0012	0.0012	0.0016	0.0016	0.0003	0.0011–0.0016
	**[30–34]**	0.0011	0.0016	0.0019	0.0025	0.0017	0.0004	0.0011–0.0024
	**35+**	0.0009	0.0014	0.0011	0.0014	0.0017	0.0003	0.0010–0.0017
Sample Size (n players)	**[19–24]**	186	196	225	225	212	819	187–230
	**[25–29]**	202	189	184	184	187	762	180–198
	**[30–34]**	92	93	82	82	84	351	80–93
	**35+**	22	18	18	18	20	78	17–21

CI: confidence interval.

### Age and years of experience

Table 6 displays the number of unique injuries and missed games when classifying players by age groups. Table 7 shows the same data when classifying the players in groups by years of experience.

**Table 7:** Injury ratios by years of experience.

	YEARS OF EXPERIENCE	2017–18	2018–19	2019–20		2020–21	Total	95% CI
				RS	Bubble			
Missed Games Ratio	**[R-5Y]**	0.0065	0.0062	0.0057	0.0039	0.0079	0.0016	0.0042–0.0079
	**[6Y-10Y]**	0.0087	0.0087	0.0104	0.0079	0.0095	0.0023	0.0079–0.0102
	**[11Y-15Y]**	0.0051	0.0079	0.009	0.0067	0.0099	0.002	0.0053–0.0101
	**16Y+**	0.0039	0.0092	0.0026	0.0053	0.0044	0.0013	0.0020–0.082
Unique Injuries Ratio	**[R-5Y]**	0.0009	0.001	0.001	0.0012	0.0013	0.0003	0.0009–0.0013
	**[6Y-10Y]**	0.0016	0.0017	0.0017	0.0025	0.002	0.0004	0.0014–0.0023
	**[11Y-15Y]**	0.001	0.0015	0.0018	0.0023	0.0018	0.0004	0.0011–0.0023
	**16Y+**	0.0011	0.0016	0.001	0.0022	0.001	0.0003	0.0007–0.0020
Sample Size (n players)	**[R-5Y]**	310	307	328	328	320	1265	306–331
	**[6Y-10Y]**	132	124	118	118	125	499	116–131
	**[11Y-15Y]**	48	54	53	53	50	205	48–55
	**16Y+**	12	12	11	11	8	43	9–13

CI: confidence interval.

### Monthly distribution

**Fig 6:**
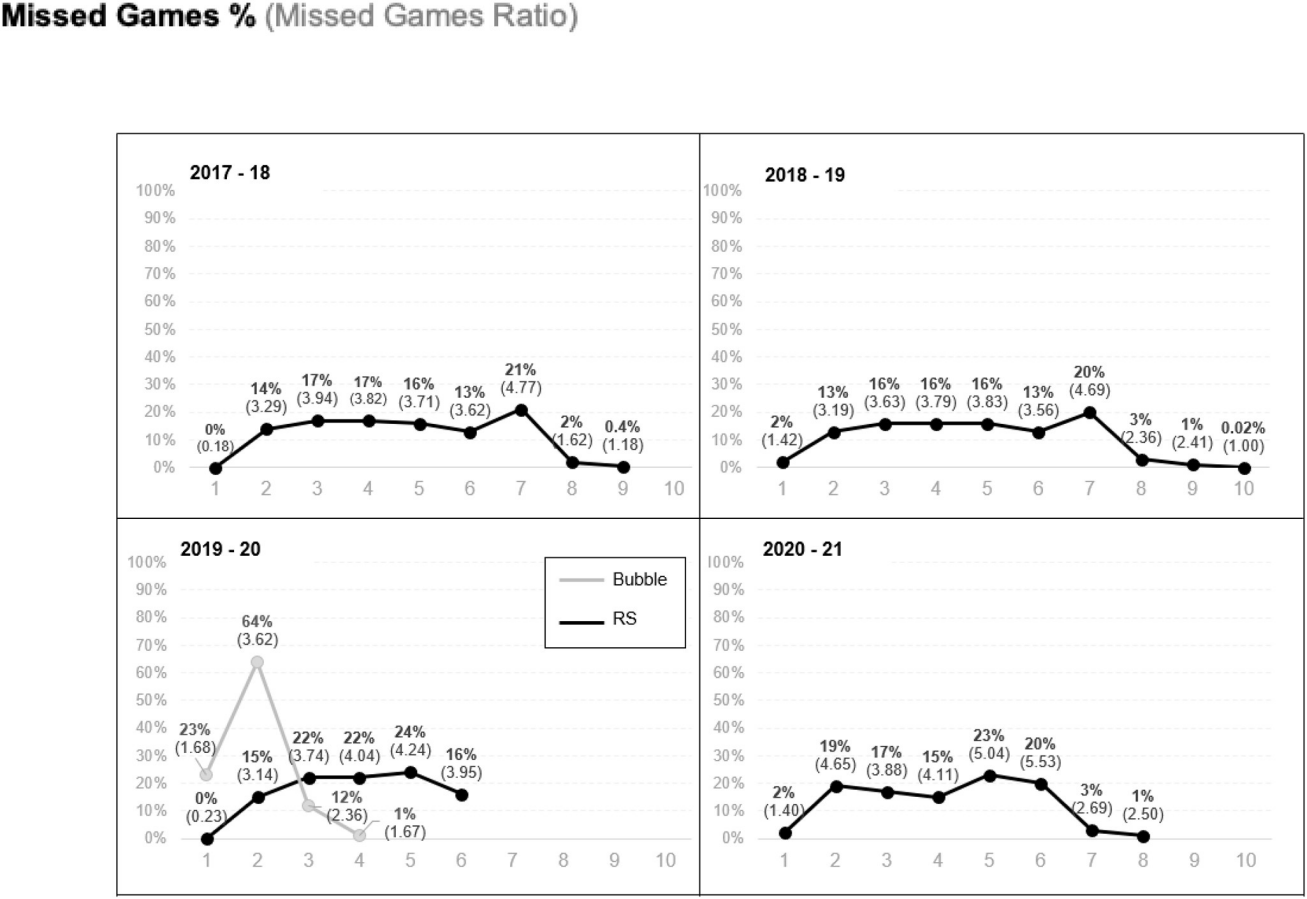
N: Number of game per team.

When analyzing the percentage and IRR of missed games by month, results showed a similar tendency, regardless of the season (Fig 6). During seasons 2017–18 and 2018–19, the higher percentage of missed games occurred in the 7^th^ month of the season, corresponding to April, representing the end of regular season or playoffs preparation, depending of the standings of the team. The Playoff phase showed a lower percentage and IRR compared to the other months of the regular season (month 1^st^ represents pre-season and friendly games). In the case of 2019–20, the highest percentage and IRR occurred in moth 5^th^ (February), before the lockdown due to COVID-19. During the Bubble, the higher percentage and IRR occurred during the 2^nd^ month (July), which was the equivalent of a regular season period. Finally, the higher percentages and IRR for 2020–21 occurred during the 5^th^ and 6^th^ month of the season (May and June), the months preceding the playoffs.

### Injuries by team

**Fig 7:**
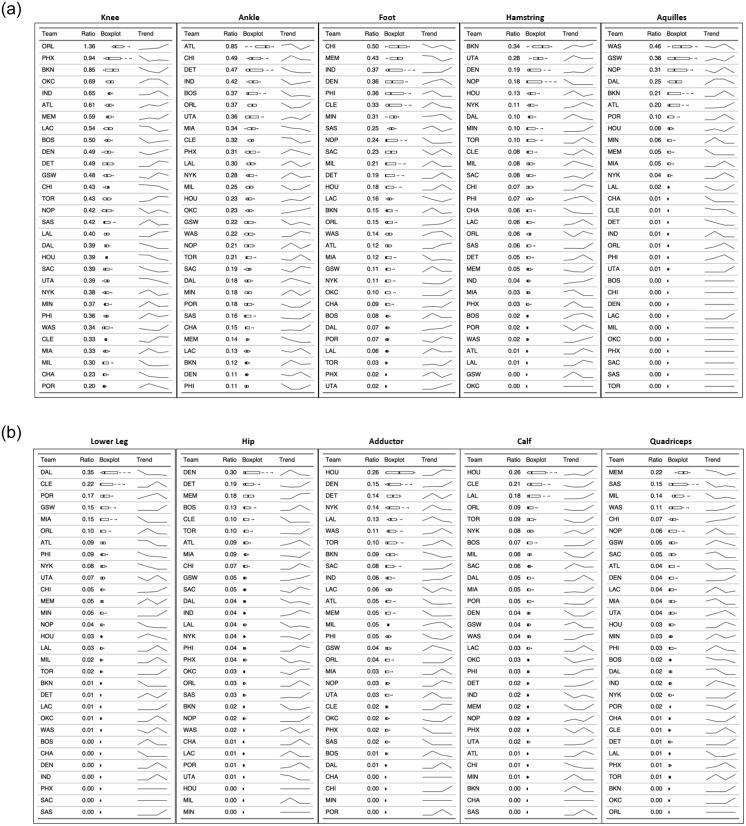
Unique injuries ratios by body area, per team, and ranked descendent.

**Fig 8:**
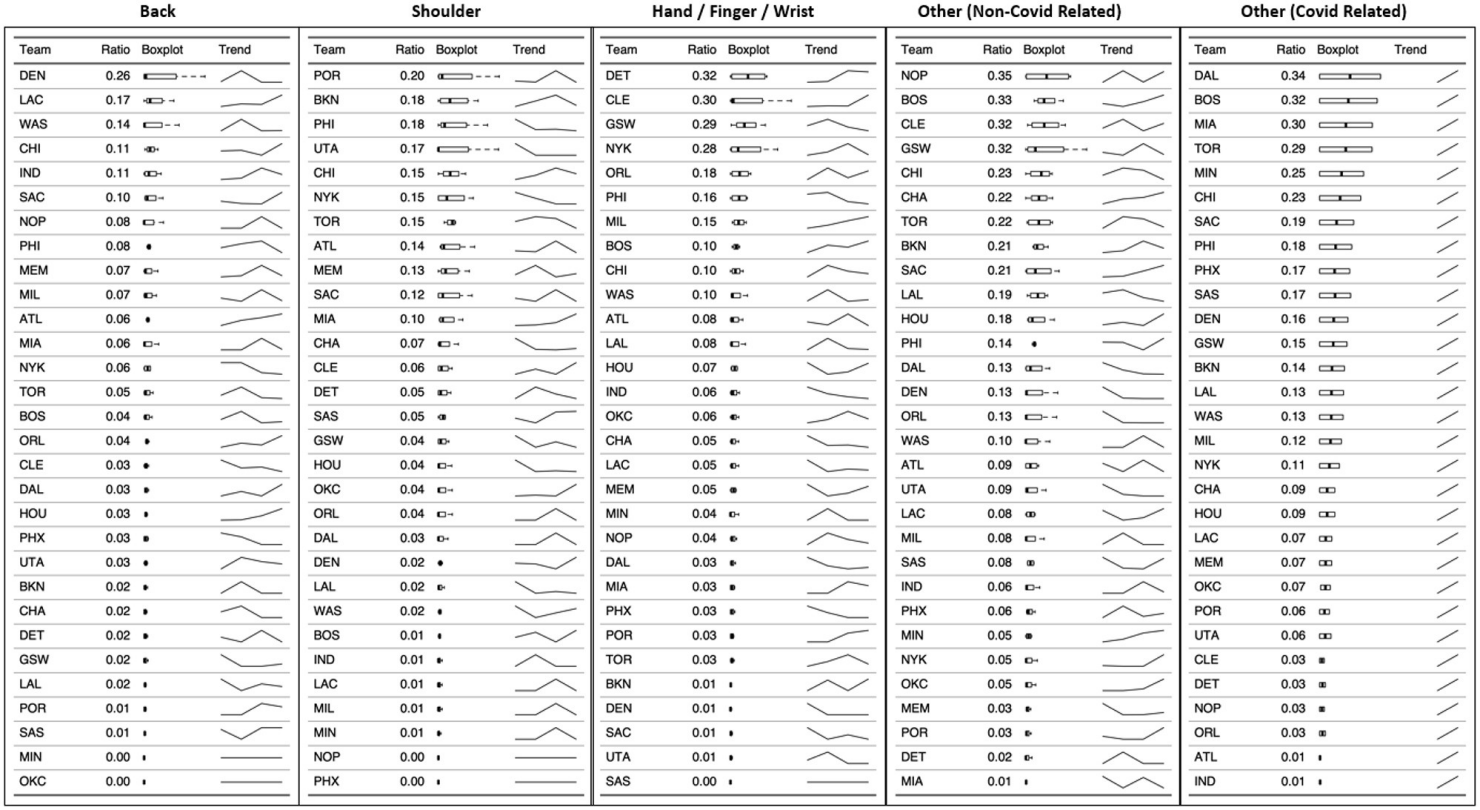
Unique injuries ratios by body area, per team, and ranked descendent.

Figs 7 and 8 display the unique injuries ratios by body area, per team, and ranked descendent. The trend line represents the evolution of unique injuries for that particular team and body area. Other unique injuries (COVID-19 related) only included two data points (2019–20 and 2020–21), while the rest of injuries included the four seasons analyzed.

## Discussion

Despite the economic impact that injuries impose on franchises, the league and broadcasters, and previous efforts to understand injury risk factors, there has been an increment of unique injuries and missed games through the period analyzed in the current study. The distribution by body area, type of injury, the time at which they occurred, minutes played and outcomes by playing position, age and years of experience varied depending on the season. Moreover, the idiosyncrasy of prevalence and incidence of injuries also varied between franchises.

As the number of unique injuries increased through the period analyzed, the number of injured players also increased. Interestingly, the number of players was consistently lower than the number of injuries, reflecting that there are a number of players that tend to re-injure. This could be explained by different reasons, including neuromuscular factors, mechanical deficits, and load considerations, among others, or by returning to the competition when they have not been fully recovered (early return to play), increasing the chances of recurrence. Analyzing the profile of the players, considering deficiencies and risk factors in training programs, effective rehabilitation programs and supervising the return to play process with objective data are critical factors avoiding recurrence.

The number of unique injuries and missed games in this study differ from previous epidemiological research in NBA players, however, comparing results with previous studies is challenging, since the databases differ. Teramoto et al. (2017) found 687 unique injuries (n = 280 unique players) from 2012–13 to 2014–15 regular seasons (3 seasons), while we found >800 unique injuries per season during the period analyzed. In our study, we found a total of 3543 number of unique injuries (4 seasons), for a total of 625 players. The 2020–21 season, corresponding to the season after a COVID-19 lockout and taking part within the context of a global pandemic, had the highest density of games in recent years. Potentially contributing to a higher number of absolute unique injuries and games missed due to injury compared to previous seasons.

### Season phase

When considering the Bubble as part of 2019–20 season, the number of unique injuries showed an incremental trend, however, when splitting the season and extracting the Bubble, results showed a decrease in both missed games and unique injuries, which means that the Bubble disrupted the incremental tendency. Some potential explanations to these results could be related to the bubble scenario, where no travel was needed, preceded by a long period of rest foregoing to the Bubble. It has been previously stated that games injuries occur more often in away games [10]. Traveling, changing time zones, jetlag, lack of recovery and sleep deprivation has been shown to decrease reaction time in athletes, and NBA schedules have also been linked to in-game injury incidence [10]. Continuing to analyze schedules, travel and time zone changes is essential to understand risk factors for injury.

### Body area

During the period analyzed, there were 697 knee, 508 ankle and 284-foot injuries representing the highest IRRs (and percentage) of injuries. In previous investigations, ankle, knee and foot have been found as the most frequently injured areas in the body, followed by other common lower body structures such as adductor/hip/groin injuries in basketball players [5, 11, 12]. Specifically in NBA players, ankle injuries have been at the top of the ranking, followed by foot, adductor/hip/groin and knee. Previous NBA epidemiological research was done prior to 2017 therefore, our results may highlight a potential change of the tendency for the most frequently injured body areas. Ankle injuries still are in the top-3 most frequently injured areas, in accordance with previous research in basketball players, and specifically in the NBA. However, adductor/hip/groin injuries have been previously reported to be on the topmost common injured areas, while in our study, the knee have been the most affected body area since the 2017–18 season, followed by ankle and foot. Nevertheless, lower body injuries, specifically knee, ankle and foot have been the most prevalent locations in recent seasons.

### Type of injury (diagnosis, mechanism)

Our results showed that the most common type of unique injury was associated to general soreness, followed by sprain, strain, medical procedures and contusions, however, when analyzing the ratio of missed games medical procedures showed the highest ratio, followed by sprain, general soreness, and strains. General soreness IRRs both for unique injuries and missed games were the highest during the Bubble, which could be expected after several weeks of lock down due to COVID-19. Sprain and strains related mechanisms have also been reported as the most common ones in previous studies. It is not surprising to see general soreness at the top of the list since 2017–18. This coincides with an increase in pace of play (possessions per game) over the last 3 seasons, reaching an all-time high since the 1988–89 season [17]. While looking into causation is beyond the scope of this work, a higher pace may contribute to elevated neuromuscular demands on players, due to being expose to a higher density of high intensity efforts (accelerations, decelerations and changes of direction) [18]. Further research into this aspect as well as the training interventions that may help mitigate this detrimental response may be important. Medical procedures and contusions are also showing higher ratios of unique injuries and missed games that in previous studies.

### Other factors

Explaining the severity (average days lost) during the period analyzed is challenging. The 2019–20 season was the shorter RS in terms of days, when excluding the Bubble period, however, due the lockout (from mid-March until July), the analysis showed this season with the higher average days lost, for both unique injuries and missed games. Hence, this was a unique context and results have to be interpreted carefully when assessing their generalizability. Despite the impact that tendon/ligament and muscle injuries might had, showing the higher ratios for both unique injuries and missed games, load-related and bone injuries kept players away for longer periods of time, missing a higher number of games. This has important implications when designing injury prevention programs, and specially rehabilitation procedures to avoid recurrence of injuries. Implementing procedures to assess and monitor players, and accounting for risk factors for these types of injuries is paramount. The average number of days lost per body area showed different trends depending on the season. Interestingly, although lower body injuries happened more frequent (higher IRRs and percentage), the average number of days lost to injury was higher in some upper body injuries. Nerve injuries showed the higher average number of days lost, followed by forearm, Achilles, arm, thoracic spine and foot.

Guards showed the highest IRR for unique injuries and missed games, for all the seasons analyzed. This result has critical implications for training programs and season management when considering playing positions, in combination with type of injuries and most common body areas injured. Taller players are usually heavier, and specific injuries might be associated to centers, such as those related to contacts or stress, while smaller players tend to be faster and perform more short high-intensity efforts, such as change of directions or jumps [1] being exposed to some of the most common type of injuries such as soreness, sprain or strains, for tendon/ligament and muscle injuries. Classifying players by position helps having a general idea of which type of players show a higher incidence of injuries, however, a deeper individual analysis is needed to be able to manage injury risk factor in different populations and type of players.

It was observed that most of both, unique injuries and missed games were concentrated in the two central experience groups (from 6 to 15 years). Players in their first 5 years in the NBA league and those who have been in the league for more than 16 years showed lower injury and missed games IRRs. This could be explained by the fact that many rookies tend to play less minutes (usually below 15 MP), and therefore, could be less exposed to risk of injury. Furthermore, the results of this study also reflected that those players playing 16–35 minutes were those with higher number of injuries and missed games. A consideration from the current results is that players with years of experience in the league (aka veterans), that haven’t been exposed to long term, traumatic or recurrent injuries, have had longer careers. Moreover, players that can sustain long careers might decrease MP, hence decreasing the changes to get overuse injuries. Players with traumatic and severe injuries between the 6^th^ and 16^th^ years of their career could have lower odds to prolong their overall career. It is worth highlighting than the afore mentioned might not apply to those players arriving in the NBA via alternative paths (i.e. players arriving from international leagues) that tend to be older and more experienced players. Regarding the age range, players between 25 to 35 years old, the age ranges where the MP per game is the highest on average, showed the highest ratios for unique injuries and missed games. We observed that the cluster of players in the 30–34 years old range also showed a higher IRR, which could be hypothetically applied to older players with a key role in their teams (e.g., starters, franchise players). These results are in accordance with previous studies where MP was significantly higher in injured players than players averaging 19.5 min per game [10]. Similarly, the assumption could be that injured players played more minutes and, thus, had a greater exposure to injury compared with uninjured athletes who played fewer minutes and, thus, had a lesser exposure to injury. However, this conclusion needs to be taken with caution. As previously stated, not every minute in the NBA weights the same. There might be younger players playing more minutes per game, with important roles in their teams, experienced players playing a high number of minutes in key games and not getting injured. Furthermore, the opponents/game difficulty, game score or factors such as playing on the road might impact the risk of injury as well [10].

In previous studies, results showed the majority of game injuries occurred before the mid-season [10], and the research suggested that NBA players transitioning into in-season games lacking the proper shape at the beginning of the season, could be affected after a long off-season period. Our results showed a different trend, where most injuries and missed games due to injury occurred from mid-season to the end of the regular season. When injuries occur in early stages of the season, Teramoto et al. (2017) reasoning, where players had long periods of off-season, could explain the results. However, for post mid-season and end of the RS injuries could be explained by other causes, such as fatigue accumulation (physical and mental), the accumulation of sleep deprivation (related to lack of recovery), or perhaps lack of competitive goals, for those teams reaching the end of the season without possibilities of making the playoffs. Moreover, playoff teams could be tapeing key players for the playoffs, overloading players with less average MP, triggering load spikes. A progression of loads during season preparation, effective training programs, managing recovery strategies during season, specially facing the end of regular season, will be paramount if the goal is to decrease injury risk.

The present study did not have as an objective to analyze the unique injuries and missed games due to injury and the competitive schedule, however, of the four seasons analyzed, 2020–21 presented a greater number of injuries, coinciding with the highest density of matches in the regular season (more RS games played in fewer total days). Previous research in NBA players population showed that playing B2B games or playing four games in five days alone was not associated with an increased rate of game injuries, whereas playing B2B games and away games were significant predictors of frequent game injuries [16]. Our results could be explained by several factors, however, a potential reason could be due to the lockout due to COVID-19, in addition to the lack of time for preparation when the 2019–20 season was resumed, the uncertainty about the schedule during the months prior to the start of the 2020–21 season, the league adjusting the number of games in a shorter period of time (starting RS in December instead of October, as previous seasons), and a season with many sanitary restrictions, mental stress and unforeseen adjustments.

### Injury trends by team

Despite there has been an increment of unique injuries and missed games through the period analyzed in the current study, the idiosyncrasies of the epidemiology and incidence of injuries varies between franchises. Some are in the top rankings in certain body area locations, while others showed practically no injuries in certain body areas. In turn, some have shown trends in reducing injuries in certain areas that are very recurrent for that franchise, while others show the opposite trend, increasing the ratios of both unique injuries and missed games. We encourage the reader is to refer to the results tables for more detail by franchise. The most important reflection in this analysis, in our opinion, is to observe which injuries present higher ratios for a given franchise and thus be able to carry out a critical analysis of the injury prevention and rehabilitation programs in that particular team.

## Limitations

This study presents certain limitations. Previous studies analyzing injuries in the NBA have used mainly two types of databases. On one hand, publicly available records and injury reports related to the NBA coming from sports media outlets (e.g., ESPN.com; CBSsports.com; NBCsports.com; FoxSports.com), basketball statistic websites (e.g., basketball-reference.com; NBAstuffer.com); NBA.com/Stats or transactional sports databases (e.g., prosportstransactions.com) [1, 2, 4, 8, 13]. On the other hand, a centralized data collection system integrated with the clinical management of player health for all NBA teams, that requires previous National Basketball Players Association (NBPA) approval to have access to the database, which represents limited access due to the lack of public access. In our study, we have used public records coming exclusively from the official NBA Box Score (PDF), which are supposed to include all the official NBA injuries reported in all played games. This dataset should be more complete than other studies using only above-mentioned public records coming from external sport media sources, as recognized for the authors in previous publications. Nonetheless, it wasn’t possible to include injuries that are registered by medical staff internally, that didn’t have to be reported for games availability, and that should be included in the NBPA database, which potentially could mean that we were missing unique injuries. Nonetheless, the players’ availability in the Box Score (PDF) is supposed to be accurate, and thus, our database should reflect the truly missed games due to injuries.

This study doesn’t include injury incidence. Injury incidence should be based on number injuries divided by the total exposure time (practice, competition) and presented as standard per 1000 h. Nonetheless, team’s practice time and schedules are not publicly reported and, therefore, only game time could be included in the calculation. As a result, the authors of the present study decided to not present injury incidence based only in game playing time. This limits the present results to only this population for the period analyzed and doesn’t allow inter-sport comparisons. Other important risk factors such as environment (where the injury occurred; training, competition, others), the cause of the injury (contact, non-contact, acute, chronic, overuse, recurrence) are not reported in the NBA Box Score report, which could help in understanding injury mechanism and help in prioritizing preventing strategies. Finally, despite the inclusion of laterality (right, left) in our database, the NBA doesn’t report players’ official laterality dominance and, therefore, the researchers of the current study have considered that analyzing the side unknowing the dominant laterality of the player does not provide relevant information. However, performing this analysis in future research is advised.

## Conclusions

Despite the economic impact that injuries supposes to franchises, the league and broadcasters, and previous efforts to understand injury risk factors, there has been an increase in unique injuries and missed games through the period of time analyzed in the current study. The distribution by body area, type of injury, when they occurred, minutes played and outcomes by play position, age or years of experience varied depending on the season. Moreover, the idiosyncrasy of prevalence and incidence of injuries also varied between franchises.

Advancing towards a better understanding of the injury landscape in the NBA will help in developing not only prevention programs at a local scale (i.e., teams, franchises) but also global injury prevention initiatives through league wide regulations, as well as to reduce the economic impact, for franchises and broadcasters, when players miss games due to injuries.
